# Rotational Spectroscopy Meets Quantum Chemistry for Analyzing Substituent Effects on Non-Covalent Interactions: The Case of the Trifluoroacetophenone-Water Complex

**DOI:** 10.3390/molecules25214899

**Published:** 2020-10-23

**Authors:** Juncheng Lei, Silvia Alessandrini, Junhua Chen, Yang Zheng, Lorenzo Spada, Qian Gou, Cristina Puzzarini, Vincenzo Barone

**Affiliations:** 1 Department of Chemistry, School of Chemistry and Chemical Engineering, Chongqing University, Daxuecheng South Rd. 55, Chongqing 401331, China; leijuncheng_1994@sina.cn (J.L.); chenjunh999@163.com (J.C.); zyzyttkx@outlook.com (Y.Z.); 2 Scuola Normale Superiore, Piazza dei Cavalieri 7, I-56126 Pisa, Italy; silvia.alessandrini@sns.it; 3 Department of Chemistry “Giacomo Ciamician”, University of Bologna, Via Selmi 2, 40126 Bologna, Italy; cristina.puzzarini@unibo.it

**Keywords:** hydrogen bond, quantum chemistry, rotational spectroscopy, noncovalent interactions, substituent effects, structure

## Abstract

The most stable isomer of the 1:1 complex formed by 2,2,2-trifluoroacetophenone and water has been characterized by combining rotational spectroscopy in supersonic expansion and state-of-the-art quantum-chemical computations. In the observed isomer, water plays the double role of proton donor and acceptor, thus forming a seven-membered ring with 2,2,2-trifluoroacetophenone. Accurate intermolecular parameters featuring one classical O-H···O hydrogen bond and one weak C-H···O hydrogen bond have been determined by means of a semi-experimental approach for equilibrium structure. Furthermore, insights on the nature of the established non-covalent interactions have been unveiled by means of different bond analyses. The comparison with the analogous complex formed by acetophenone with water points out the remarkable role played by fluorine atoms in tuning non-covalent interactions.

## 1. Introduction

The presence of different chemical groups in a given molecular system leads to a number of possible binding sites, which might dramatically tune the intra- or inter-molecular interaction topologies due to their bond donor and/or acceptor characteristics. Intra-molecular non-covalent interactions (NCIs) among different moieties can indeed favor specific conformers (see for example [1,2]), whereas inter-molecular NCIs can tune the preference among different supramolecular architectures [3,4], such interactions being indeed at the basis of molecular recognition mechanisms. In this respect, the specificity of NCI-mediated mechanisms has been recently exploited to determine the absolute configuration of a selected enantiomer by introducing the so-called “chiral tag” method [5]. Among the possible interactions, hydrogen bond (HB) [6,7] is widespread in nature, frequently playing a fundamental role in life processes, e.g., by tuning nucleobase pairing in DNA and RNA, or in self-assembling processes in functional materials [8], organocatalysis [9] and many other situations. In this respect, HB features can be characterized by quantum-chemical methods [10,11,12] as well as by different spectroscopic techniques, such as the X-ray [13,14], NMR [14,15], IR [14,16,17,18], Raman [17,18], ZEKE [19], REMPI [19] and microwave (MW) [20,21] spectroscopy. Indeed, the combination of this last technique with supersonic expansion makes it possible to generate stable weakly bound complexes in the gas phase that can be characterized by means of their rotational spectra. MW spectroscopy, when integrated with quantum chemistry, allows for unveiling accurate structural parameters without the perturbing effect of solvent or matrix environments and, ultimately, permits the intrinsic structural and dynamical factors governing the observed NCIs to be disclosed.

A detailed knowledge of the HB strength and directionality for different binding sites and their comparison to other NCIs is of fundamental interest. In fact, their characterization can provide insights into the design of new “molecular bricks” with specific functions or, more simply, on the variety of isomeric structures present in different environments for a given molecular system. The investigation of the change of conformational or, more generally, isomeric equilibria when going from gas to condensed phases allows the disentanglement of the role of intrinsic and environmental effects in tuning the structures and properties of flexible molecules of biological interest. Particularly interesting chemical properties, significantly affecting not only the NCIs topology, but also the electronic features of the molecule itself, are encountered when the H→F substitution is introduced, whose effect is progressively more significant as the number of fluorine atoms increases [22]. The effects of fluorination on NCIs can be addressed by focusing on the prototype aromatic compound, i.e., benzene [23], its mono-/di-fluorinated derivatives [24], and its fully fluorinated analogous [25] and using water (W) as the molecular probe. In fact, rotational spectroscopy investigations unequivocally proved that, while benzene forms an O-H∙∙∙π HB bound complex [23] with water, a six-membered planar (or nearly planar) structure, involving O-H···F and C-H···O NCIs, is established in both 1:1 fluorobenzene∙∙∙water and *p*-difluorobenzene∙∙∙water adducts [24], whereas the lone pair (O)∙∙∙π-hole linkage governs the intermolecular bonding in the hexafluorobenzene∙∙∙water complex [25].

Among different fluorinated moieties, the trifluoromethyl group is a particularly interesting case because it shows unique and distinctive properties [26,27,28], whose importance is witnessed by its incorporation in many drugs. Several studies have shown that trifluorination of the methyl group has a significant effect on the conformational preference of a molecule. In particular, microwave investigations of trifluoroanisole (PhOCF_3_) [29] and anisole (PhOCH_3_) [30,31] have pointed out that, while in the former case the -CF_3_ moiety prefers an orthogonal conformation with respect to the phenyl ring, in the latter system the -CH_3_ group adopts a planar arrangement with respect to the aromatic ring. The same conformational behavior has been also evidenced in O→S substituted compounds, e.g., trifluorothioanisole (PhSCF_3_) [32] and thioanisole (PhSCH_3_) [33], thus suggesting that the conformational preference is unaffected by chalcogen substitution, and only depends on the H→F substitution effect. Another interesting fluorination effect is highlighted by the shapes of microsolvated (water molecule) anisole and trifluoroanisole. In fact, although the monomers have the same conformational backbone structure in the complexes and in the isolated molecules, in the case of the former, water forms one bifurcated O-H···O HB, above and below the anisole plane, together with one C-H···O HB, whereas in the trifluoroanisole-water complex O-H···O and C-H···O HBs are established within the phenyl ring plane. Moving from -XCY_3_ (with X = O or S, Y = H or F) to –C = OCY_3_ (Y = H or F) as phenyl substituent, no trifluomethylation effect on the conformational preference is observed, as revealed by gas-phase investigations of acetophenone (AP) [34,35] and its trifluoromethyl analogue, 2,2,2-trifluoroacetophenone (TFAP) [36], both showing planar backbone structures.

With the aim of further elucidating the effect of fluorination in prototypical complexes, we undertook a combined experimental-theoretical study, involving rotational spectroscopy in supersonic expansion and state-of-the-art quantum-chemical calculations, of the 1:1 adduct formed by TFAP and water. This work has three main goals:

1. To investigate whether trifluoromethylation plays a role in tuning the NCIs established by TFAP and water with respect to the 1:1 AP-water complex [35].
2. To accurately characterize the intermolecular parameters by means of a semi-experimental approach for the equilibrium structure determination applied to the TFAP-water complex [37,38,39].
3. To describe the interactions and the contributions involved in the stabilization of the complex using different quantum-chemical energy partitioning techniques.

## 2. Results and Discussion

### 2.1. Structures and Energetics of the Low-Lying Isomers

**Figure 1:**
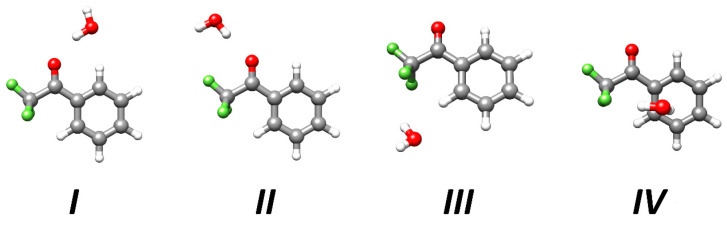
Molecular structures of the low-energy isomers (numbered according to the relative stability) of the TFAP-W complex issuing from rDSDjun computations.

Figure 1 sketches the structures of the four isomeric species (selected according to the strength of HBs between water and TFAP) that have been optimized at the level denoted as rDSDjun (see Section 3.2 for its definition), and reported in Table S1 of the Supplementary Materials. The corresponding (straightforwardly derived) equilibrium rotational constants and the relative stabilities, obtained by means of the so-called CP-jChS approach (see Section 3.2 for its definition), are collected in Table 1. The isomers are labelled according to their relative stability (see Figure 1), and their shapes are determined by the different HB linkages that water can establish with three specific moieties of TFAP, i.e., the carbonyl and -CF_3_ groups as well as the aromatic ring. These moieties can form different kinds of HBs with water, namely the O-H∙∙∙O, C-H∙∙∙O, O-H∙∙∙F and O-H∙∙∙π interactions, with the O-H∙∙∙O HB being typically much stronger than the others.

Since water can form two non-equivalent O-H∙∙∙O HBs with the carbonyl oxygen lone pairs of TFAP, a non-negligible role is likely played by secondary interactions such as the C-H∙∙∙O HB in the case of isomer *I*. While a detailed analysis on the reason why isomer *I* is more stable than isomer *II* is provided in Section 2.5 (see below), the two less stable structures, namely isomers *III* and *IV*, owe their higher energy to the absence of any O-H∙∙∙O HB linkage. Furthermore, the formation of the C-H∙∙∙O and O-H∙∙∙F contacts favors isomer *III* with respect to isomer *IV*, which is stabilized by O-H∙∙∙F and O-H∙∙∙π interactions. Focusing the attention on the most stable (by far) complex structure (isomer *I*, see Table 1), at the rDSDjun level, the frequency of the lowest normal mode (associated with the out-of-plane motion of the water molecule) is very small, thus suggesting that the experimentally observable structure is effectively planar (as obtained at the level of theory defined as B3; for its definition, see Section 3.2) and, consequently, a *C*_s_ symmetry was enforced for isomer *I* in all calculations.

### 2.2. Rotational Spectroscopy Investigation

According to the quantum-chemical predictions reported in Table 1, the electric dipole moment component along the *a*-inertial axis (*μ*_a_) is the largest one except for isomer *III*. As a consequence, the initial spectral survey aimed at searching for and assigning *μ*_a_-*R*-type transitions of the most stable species (i.e., isomer *I*). Thanks to the theoretical predictions, a total of more than one hundred *μ*_a_- and *μ*_b_-type rotational transitions belonging to isomer *I* have been observed. The assignment to this specific isomer is confirmed by the good agreement between the computed and experimental rotational (the largest discrepancy being 0.7%) and quartic centrifugal distortion constants (see Table 2). In a second step, the rotational spectra of isomer *I* formed with the H_2_^18^O, HOD, DOH, and D_2_O water isotopologues were measured, further confirming the correct assignment of the isomeric species. All measured frequency values are reported in the Supplementary Materials (Tables S2–S6).

**Figure 2:**
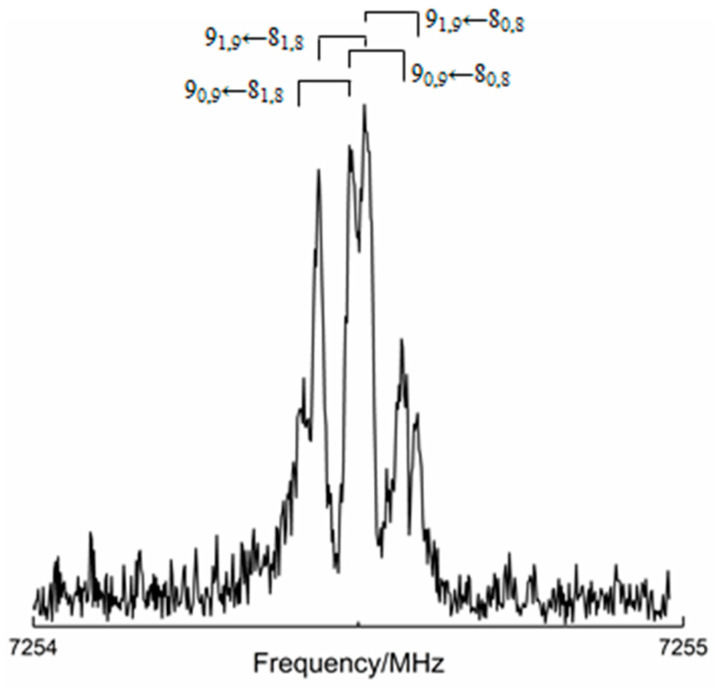
Four rotational transitions (labels: *J_Ka,Kc_*, *J* being the rotational quantum number and *K_a_*, *K_c_* the values of the quantum number *K* in the prolate and oblate limiting case, respectively) of the main isotopologue of the isomer *I* of TFAP-W.

An example of a small portion of the recorded spectrum for the main isotopic species of isomer *I* is given in Figure 2. All the retrieved transition frequencies have been fitted using Pickett’s SPFIT program [40] within the semi-rigid Watson’s Hamiltonian (*S* reduction; *III*^l^ representation) [41]. For all the isotopologues investigated, the experimental spectroscopic parameters are collected in Table 2 where, as already mentioned, they are compared—for the main isotopic species—with the corresponding theoretical values (see Section 3.2). The planarity (or near planarity) of the observed molecular complex can be deduced from the comparison of the planar moments of inertia along the *c*-axis (*P*_cc_) of the various isotopologues, which are reported in Table 2, with that of the isolated TFAP (45.01450(7) uÅ^2^) [36]. In fact, since *P*_cc_ represents the mass extension along the *c*-axis, which is perpendicular to the *ab*-plane (the plane where the complex lies), the similarity of its value for all isotopologues and the isolated TFAP suggests that the presence of water does not produce any significant variation along the *c*-axis, thus preserving the *C*_s_ symmetry of the isolated monomer in the molecular complex.

### 2.3. Semi-Experimental Equilibrium Structure

**Figure 3:**
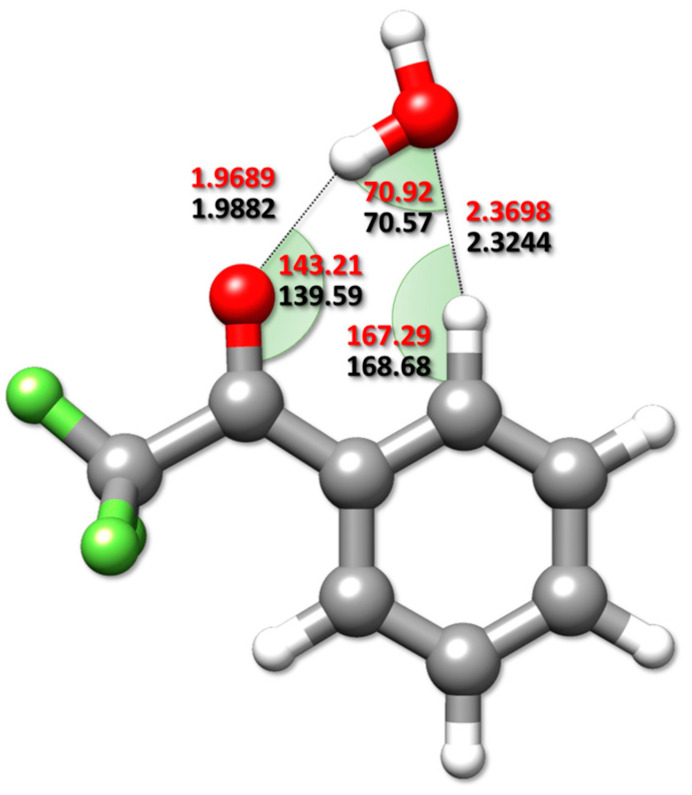
Comparison between the semi-experimental intermolecular parameters (in red) with the rDSDjun counterparts (in black). Distances and angles are given in Å and degrees, respectively.

Thanks to the availability of experimental rotational constants for different isotopic species of the observed complex (isomer *I*), together with the semi-experimental equilibrium structures of the two monomers (see Section 3.2.2), accurate semi-experimental equilibrium intermolecular parameters have been obtained for the TFAP-W complex. Namely, the O_water_···O_TFAP_ distance and the O_water_···O_TFAP_ = C angle (2.8848(4) Å and 136.08(2)°, respectively, with σ^2^ = 0.05) have been determined employing our molecular structure refinement (MSR) software [42]. The derived intermolecular parameters are compared in Figure 3 with the rDSDjun counterparts. A reasonably good agreement is noted, even if a different trend for the distances describing the established NCIs is evident for $r_{e}^{SE}$ with respect to the rDSDjun level. In fact, going from $r_{e}^{SE}\;$ to rDSDjun, a lengthening of the O-H∙∙∙O contact (of about 0.02 Å) and a shortening of the C-H∙∙∙O weak hydrogen bond (wHB) (of about 0.05 Å) are observed. A noticeable difference has also been found for the C = O∙∙∙H_water_ angle (~4°), while the C-H∙∙∙O angle and that defining the orientation of water within the plane of symmetry of the complex show deviations of 1.4° and 0.4°, respectively.

### 2.4. Bond Analysis

The nature of the NCIs (HBs in the present case) stabilizing the observed complex has been unveiled by combining different types of bond analyses (whose acronyms and details are provided in Section 3.2.3), thus providing a full description of these linkages. The detailed results can be found in the Supplementary Materials (Tables S7 and S8).

**Figure 4:**
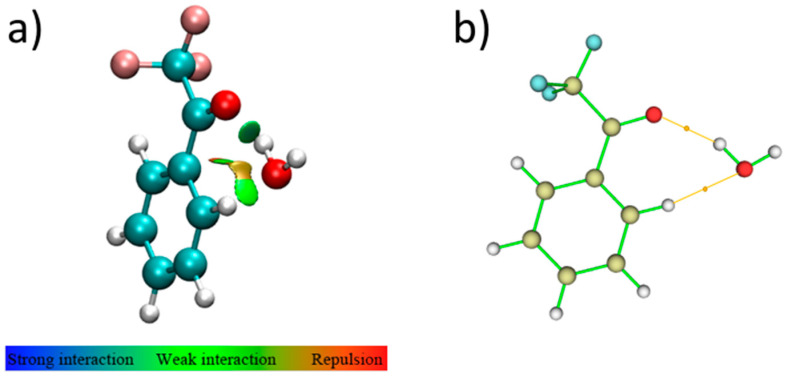
(**a**) Noncovalent interactions stabilizing the isomer *I* of TFAP-W. (**b**) QTAIM analysis of isomer *I*. The bond critical points (i.e., the saddle points of electron density between two atoms) are identified by orange dots and the bond paths are represented by orange lines.

The observed molecular complex is characterized by a seven-membered ring in which one O-H···O HB and one C-H···O wHB are established between TFAP and water. The former is a classical HB, stronger than the latter one, as shown in Figure 4a, which graphically reports the results of the QTAIM analysis: O-H∙∙∙O −21.9 kJ·mol^−1^ vs. C-H∙∙∙O −8.6 kJ·mol^−1^. These results are also in agreement with the outcome of the NBO analysis; in fact, the E(2) contributions between occupied (bonding (BD) or lone-pair (LP)) and empty (antibonding (BD*)) localized orbitals larger than 1.0 kJ·mol^−1^ are:

(1) LP(1) O_C=O_-BD*(1) O-H_HB,water_: 10.0 kJ·mol^−1^, and LP(2) O_C=O_-BD*(1) O-H_HB,water_: 10.8 kJ·mol^−1^ vs. LP(2) O_water_-BD*(1) C-H_TFAP_: 4.9 kJ·mol^−1^;
(2) BD(1) O-H_HB,water_-BD*(1) C-H_TFAP_: 1.1 kJ·mol^−1^.

The comparison of the NBO charges in the isolated TFAP and water monomers with the results for the isomer *I* of the TFAP-W complex (see Table S7) suggests a small charge rearrangement of the atoms involved in the HBs, whereas the charges of the -CF_3_ group and part of the benzene ring are not affected by the interaction with the water molecule.

The SAPT analysis (see Table S8) points out that the electrostatic term (−32.4 kJ·mol^−1^) is the largest stabilizing interaction, while dispersion (−14.9 kJ·mol^−1^) and induction (−10.7 kJ·mol^−1^) are comparable. These three contributions overcome the exchange term (36.8 kJ·mol^−1^), thus resulting in a total energy of −21.2 kJ·mol^−1^, which is in good agreement with the CP-jChS equilibrium dissociation energy (*D*_e_) of −22.4 kJ·mol^−1^ (with the Δ^def^ term being 0.9 kJ·mol^−1^; for its definition, see Section 3.2).

### 2.5. 2,2,2-Trifluoroacetophenone vs. Acetophenone: Effect of Fluorination on the Complex Structure

The study of both the AP-W [35] and TFAP-W complexes allows a detailed analysis of the changes produced by the -CH_3_→-CF_3_ substitution on the characteristics of intermolecular HBs. Since the electrostatic contribution is the largest term stabilizing the interaction with water in the global minima of both isomers *I* of the TFAP-W (for SAPT, see above) and AP-W (SAPT: Electrostatic −44.0 kJ·mol^−1^, Induction −15.7 kJ·mol^−1^, Dispersion −16.2 kJ·mol^−1^) complexes, and the total interaction energies are of the same order of magnitude (TFAP-W −21.2 vs. AP-W −25.9 kJ·mol^−1^), an investigation of the electrostatic potential of the isolated monomers can provide useful insights. In this respect, according to the MP2/6-311++G(d,p) electrostatic surface potentials of AP and TFAP, shown in ref. [36], the -CH_3_→-CF_3_ substitution produces three main effects:

1. The aromatic C-H groups present a more positive electrostatic potential.
2. The electron density above the aromatic ring and on the outer carbonyl oxygen is reduced.
3. The positive electrostatic potential around the -CH_3_ group in AP becomes negative around the -CF_3_ group in TFAP.

**Figure 5:**
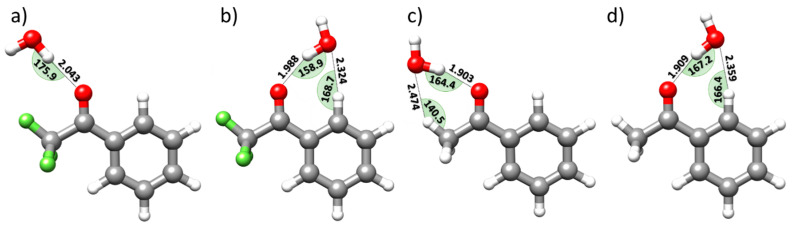
rDSDjun structures of the isomer *II* (**a**) and isomer *I* (**b**) of TFAP-W and of the isomer *I* (**c**) and isomer *II* (**d**) of AP-W. The parameters involved in the corresponding HBs are also reported (bonds are expressed in Å and angles in degrees).

According to these effects and with reference to Figure 5 (which sketches the structures of the two most stable isomers for both TFAP-W and AP-W), interesting insights can be gained on water preferences in linking TFAP and AP. First, focusing on the TFAP-W complex, it is important to investigate why isomer *II* (Figure 5a) is less stable than Isomer *I* (Figure 5b). At first glance, both show similar O-H···O HBs. However, their lengths (2.043 Å vs. 1.988 Å) suggest that the O-H···O HB in isomer *I* is stronger than that in isomer *II*, even though isomer *II* shows a more linear linkage (175.9° vs. 158.9°). This reasoning is also confirmed by the NBO analysis (E(2) contributions ≥ 1.0 kJ·mol^−1^: LP(1) O_C=O_-BD*(1) O-H_HB,water_ and LP(2) O_C=O_-BD*(1) O-H_HB,water_ are 6.9 and 9.0 kJ·mol^−1^, respectively, for Isomer *II* vs. 10.0 and 10.8 kJ·mol^−1^ for isomer *I*). Furthermore, while in isomer *I* a C-H···O wHB is the established (the total E(2) term being larger than 6 kJ·mol^−1^), in Isomer *II*, the angle between O-H (water) and the closest fluorine atom being about 105° hampers the formation of a secondary O-H···F wHB.

Concerning AP-W [35], it is clear that both the isomers shown in Figure 5c,d are engaged in classical O-H···O HBs stronger than that present in the Isomer *I* of TFAP-W (Figure 5b), as evidenced by the corresponding interatomic distances and angles. This is also confirmed by the NBO analysis (LP(1)O_C=O_-BD*(1) O – H_HB_,_water_ and LP (2) O_C=O_-BD*(1) O-H_HB_,_water_ are: 9.7 and 28.7 kJ·mol^−1^, and 14.6 and 18.5 kJ·mol^−1^ for the isomers *I* and *II* of AP-W, respectively, vs. 10.0 and 10.8 kJ·mol^−1^ for the isomer *I* of TFAP-W). However, while the Isomer *I* of TFAP-W (Figure 5b) shows a C(sp^2^)-H···O wHB, the isomer *I* of the AP-W complex (Figure 5c) presents a C(sp^3^)-H···O wHB [35]. The corresponding geometrical parameters (namely, the (C)-H···O distances and the C-H···O angles) reflect the relative strength of the corresponding wHBs, in agreement with the results of the NBO analysis (see Supplementary Materials). In fact, as highlighted in Figure 5b,c, the C-H···O wHB in the isomer *I* of TFAP-W is about 0.15 Å shorter than that in the isomer *I* of AP-W, this latter showing a C-H···O angle that deviates from the linearity more than in the isomer *I* of TFAP-W (140.5° vs. 168.7°). While both the isomer *I* of TFAP-W (Figure 5b) and the isomer *II* of AP-W (Figure 5d) show a seven-membered ring, a six-membered ring is observed in the isomer *I* of AP-W (Figure 5c). On the other hand, the structural and NBO parameters (see Supplementary Materials for a full account) point out the greater strength of the C-H···O wHB in the former complex, in agreement with the C-H electron density reduction in TFAP with respect to AP. The most remarkable intermolecular parameters of the TFAP-W complex (see Figure 5b) are the C-H···O distance (2.324 Å) and the associated angle (168.7°), to be compared with the corresponding parameters in the second most stable isomer of AP-W (Figure 5d), namely a C-H···O distance of 2.359 Å and an angle of 166.4°. In the same vein, the E(2) contributions ≥ 1.0 kJ·mol^−1^ for the observed TFAP-W complex are: LP(2) O_water_-BD*(1) C-H_TFAP_ (4.9 kJ·mol^−1^) and BD(1) O-H_HB,water_-BD*(1) C-H_TFAP_ (1.1 kJ·mol^−1^). The leading E(2) contributions in the second most stable isomer of the AP-W complex are instead: LP(2) O_water_-BD*(1) C-H_AP_ (3.6 kJ·mol^−1^) and BD(1) O-H_HB,water_-BD*(1) C-H_AP_ (1.2 kJ·mol^−1^).

## 3. Materials and Methods

### 3.1. Experimental Details

The TFAP-H_2_O complex was produced in the supersonic expansion under optimized conditions. Rotational spectra were measured using the highly integrated pulsed jet Fourier-Transform MW (FTMW) spectrometer [43], equipped with a coaxially oriented beam-resonator arrangement, COBRA-type [44], at Chongqing University [45], which covers the 2.0–20.0 GHz frequency range. Each rotational transition appears as a doublet due to the Doppler effect, which is a consequence of the coaxial arrangement between the resonator and the supersonic jet. Therefore, each rest frequency was estimated from the arithmetic mean of the two Doppler components. For the present study, the estimated accuracy of the frequency measurements is 3 kHz.

From the operational point of view, the helium buffer gas at a stagnation pressure of ~0.1 MPa was passed over a mixture of TFAP (commercial sample from Macklin (Shanghai, China) used without further purification, heated up to 313 K) and water (or H_2_^18^O or D_2_O), and expanded through the solenoid valve (Parker-General Valve, Series 9, nozzle diameter 0.5 mm) into the Fabry-Pérot cavity (~10^−5^ Pa).

### 3.2. Theoretical Methodology

#### 3.2.1. Isomers Characterization

The search for low energy isomers has been performed using the B3LYP hybrid density functional [46,47,48], augmented by the D3(BJ) [49,50] dispersions correction and in conjunction with the double-zeta basis set developed at Scuola Normale Superiore (SNSD) [51,52]. Overall, this level of theory has been denoted as B3. More accurate structures and harmonic zero-point energies of the low-lying energy minima have then been obtained using the double-hybrid revDSD-PBEP86-D3(BJ) functional [53] in conjunction with the jun-cc-pVTZ (jTZ) basis set [54] (this level of theory has been denoted as rDSDjun). From a spectroscopic point of view, while equilibrium geometries straightforwardly provide equilibrium rotational constants, the prediction of the vibrational ground-state rotational constants requires the incorporation of vibrational corrections, which have been obtained from anharmonic force field calculations at the B3 level (for a detailed account, see e.g., ref. 39). As mentioned in the caption of Table 1, these computations also provided the quartic centrifugal distortion constants. Finally, the dipole moment components needed for estimating the intensity of rotational transitions have been obtained at the rDSDjun level.

On top of the rDSDjun-optimized geometries, improved electronic energies have been evaluated by applying the so-called “jun-cheap” composite scheme (jChS) [11], also including the counterpoise correction (CP) to recover the basis set superposition error [55] (CP-jChS). The jChS model (see Equation (1)), which has been purposely developed for molecular complexes [11], starts from coupled-cluster computations including single and double excitations augmented by a perturbative estimate of triples, CCSD(T) [56], in conjunction with the jTZ basis set and within the frozen-core (fc) approximation. To improve this level of theory, the jChS model considers the extrapolation to the complete basis set (CBS) limit and the effect of core-valence (CV) correlation using Møller-Plesset theory to second order (MP2) [57]:

(1) $$E^{jChS}=\;E\left(CCSD\left(T\right)/jTZ\right)+∆E^{MP2/\infty }+\Delta E_{CV}^{MP2/CTZ}$$

where

(2) $$∆E^{MP2/\infty }\;=\;\frac{Y^{3}∆E^{MP2/jYZ}-X^{3}∆E^{MP2/jXZ}}{Y^{3}-X^{3}}-E^{MP2/jXZ}$$

(3) $$\Delta E_{CV}^{MP2/CTZ}\;=\;E^{ae-MP2/CTZ}-E^{fc-MP2/CTZ}$$

In the above expressions, $∆E^{MP2/\infty }$ is obtained by extrapolating to the CBS limit the fc-MP2 total energies obtained in conjunction with the jTZ (*X* = 3) and jQZ (*Y* = 4) basis sets using the *n*^−3^ formula by Helgaker and coworkers [58], thus extrapolating in a single step both the HF and MP2 contributions. $\Delta E_{CV}^{MP2/CTZ}$ represents the CV correlation correction derived as the difference between the MP2 energy evaluated correlating all electrons (ae) and that computed within the fc approximation, both in conjunction with the cc-pwCVTZ (CTZ) basis set [59].

The corresponding equilibrium dissociation (*D*_e_) and interaction ($E_{CP}^{int}$) energies are connected by the relationship *D*_e_ = $E_{CP}^{int}$ − Δ^def^, with the last term (Δ^def^) being the corresponding deformation contribution [11].

All the calculations have been performed using the Gaussian package (G16.C01 release [60]).

#### 3.2.2. Semi-Experimental Equilibrium Structure

Currently, one of the most accurate ways to determine equilibrium structures is represented by the so-called semi-experimental approach [37,38,39]. This strategy consists in performing a least-squares fit procedure to derive the structural parameters from the semi-experimental equilibrium rotational constants ($B_{e}^{i}$, with *i* = *a*,*b*,*c*), which are obtained by subtracting from the available experimental ground-state rotational constants ($B_{0,exp}^{i}$) the corresponding computed vibrational contributions ($∆B_{vib}^{i}$):

(4) $$B_{e}^{i}=B_{0,exp}^{i}-∆B_{vib}^{i}$$

In this study, as briefly mentioned in the previous section, the $∆B_{vib}^{i}$’s have been evaluated at the B3 level in the framework of second-order vibrational perturbation theory (VPT2) [61,62]. A possible additional correction is provided by the electronic contribution to the rotational constants, which is, however, usually negligible [39,63].

Unfortunately, the large number of isotopic species required to correctly determine all structural parameters of a molecular system at equilibrium is rarely available. In this respect, a reliable way-out to overcome the lack of experimental data without a significant loss of accuracy is represented by the so-called “template approach” [39,63]. In such model, the non-determinable parameters are fixed to the corresponding computed values corrected by the computed experimental difference for a known reference structure (template molecule). Within this framework, the semi-experimental equilibrium structure ($r_{e}^{SE}$) of the observed TFAP-W complex has been obtained in two steps:

(1) Use of the available semi-experimental structure of isolated water [39,63] and determination of the semi-experimental equilibrium structure for the isolated TFAP monomer.
(2) Determination of the semi-experimental equilibrium structure of the TFAP-W complex by using the “template approach” to fix the internal coordinates of the two partners in the complex, and fitting only the inter-molecular parameters.

Concerning the first step, thanks to the availability of the vibrational ground-state rotational constants of TFAP [36] for the most abundant species and for all 8 monosubstituted-^13^C isotopologues, the $r_{e}^{SE}$ of the isolated monomer has been evaluated by fitting the carbon backbone structural parameters while keeping fixed at the rDSDjun values the remaining internal parameters. Then, the intra-molecular parameters of the TFAP-W complex ($r_{e}^{SE\left(intra\right)})$ have been obtained by correcting the rDSDjun optimized parameters according the following equation:

(5) $$r_{e}^{SE\left(intra\right)}\;=\;r_{e}^{rDSDjun\left(TFAP-W\right)}\;+\;(r_{e}^{SE\left(TFAP/H2O\right)}\;-\;r_{e}^{rDSDjun\left(TFAP/H2O\right)})$$

where the template correction for TFAP or water (second term of the right hand-side) is applied according to the monomer under consideration. Finally, the semi-experimental structure of the complex ($r_{e}^{SE\left(TFAP-W\right)})$ has been obtained by fitting only the selected intermolecular parameters mentioned above.

#### 3.2.3. Bond Analysis

Four different types of bond analysis have been performed, namely Natural Bond Orbital (NBO) [64], the Symmetry Adapted Perturbation Theory (SAPT) [65], the Non Covalent interaction Index (NCI) [66] and the Quantum Theory of Atoms In Molecules (QTAIM) [67]. The NBO analysis employs localized orbitals and second-order perturbation theory to compute the corresponding interaction energies (E2), whereas SAPT employs inter-molecular perturbation theory to determine the different contributions (electrostatic, exchange, induction, dispersion) to the interaction energy between the partners of a complex. The NCI analysis identifies the interactions in chemical systems on the basis of the electron density and its derivatives, while QTAIM is based on the identification of the basins of the electron density distribution function and the location of the corresponding critical (especially saddle) points. From technical point of view, the NBO analysis has been performed using the G16 program [60] at the B3LYP-D3(BJ) level in conjunction with the maug-cc-pVTZ-*d*H basis set [68] and employing geometries optimized at the same level of theory. All the other analyses have been performed using rDSDjun reference geometries: the SAPT analysis (at the SAPT2+3(CCD)/aug-cc-pVDZ-RI level) with the PSI4 program [69], the NCI and QTAIM analyses with the Multiwfn program [70] (the VMD software [71] being used for visualization).

## 4. Conclusions

The hydrogen bonding features involved in the formation of the TFAP-W complex have been unveiled by combining rotational spectroscopy in supersonic expansion with state-of-the-art quantum-chemical calculations. In the experimentally observed isomer (*I*), TFAP and water are linked by one O-H···O HB and one C-H···O wHB, thus leading to an estimated equilibrium dissociation energy of −22.4 kJ·mol^−1^, with the electrostatic term being the largest contribution stabilizing the complex. The role of C-H···O wHBs in assisting stronger intermolecular interactions was already pointed out in the case of the cyclopentene-water complex [72], where the main interaction is O-H···π [72].

Accurate equilibrium values of the key intermolecular parameters have also been derived in the framework of the semi-experimental approach. The comparison of the structures of the most stable water complexes formed by AP and TFAP points out that the -CH_3_→-CF_3_ substitution favors the formation of a seven-membered ring involving a (CH)_aromatic_···O wHB, in contrast to the six-membered ring of AP-W [35], involving one H_2_C-H···O wHB.

## Figures and Tables

**Table 1:** Equilibrium rotational constants (MHz) ^1^, electric dipole moment components (debye) ^2^, relative equilibrium ^3^ and zero-point corrected ^4^ energies (kJ·mol^−1^), equilibrium dissociation and interaction energies (kJ·mol^−1^) ^5^ of the TFAP-H_2_O isomers.

	*I*	*II*	*III*	*IV*
*A_e_*	891.23	1277.58	1027.75	1193.21
*B_e_*	611.55	472.14	603.12	520.70
*C_e_*	387.60	367.15	408.87	483.30
|*μ*_a_|	1.8	5.5	1.5	2.6
|*μ*_b_|	1.7	2.2	2.1	0.7
|*μ*_c_|	0.0	0.0	0.5	2.5
Δ*E*	0.0	5.0	9.3	12.6
Δ*E*_0_	0.0	4.5	6.9	9.4
*D*_e_	−22.36	−17.29	−13.11	−9.96
$E_{CP}^{int}$	−23.24	−17.56	−13.62	−10.65

^1^*A_e_*, *B_e_* and *C_e_*: at the rDSDjun level. ^2^ *μ*_a_, *μ*_b_ and *μ*_c_: absolute equilibrium rDSDjun values. ^3^ CP-jChS relative equilibrium energies (Δ*E*). ^4^ CP-jChS relative equilibrium energies zero-point corrected at the rDSDjun level (Δ*E*_0_). ^5^ CP-jChS equilibrium dissociation (*D*_e_) and interaction energies ($E_{CP}^{int}$); for their definition, see Section 3.2.

**Table 2:** Experimental spectroscopic parameters of different isotopic species of the TFAP-W isomer *I* (*S*-reduction, *III* ^l^ representation).

	Theory ^1,2^	TFAP-H_2_O	TFAP-H_2_^18^O	TFAP-D_2_O	TFAP-HOD	TFAP-DOH
*A*_0_/MHz	879.36	878.0858(1) ^3^	833.9848(2)	830.3953(1)	845.3957(1)	861.6862(1)
*B*_0_/MHz	605.16	609.4679(1)	607.227(1)	608.7236(6)	608.6732(9)	609.5202(9)
*C*_0_/MHz	383.35	384.50355(4)	374.9407(1)	374.82029(6)	377.82212(9)	381.3495(1)
*D*_J_/kHz	0.09	0.121(1)	0.117(2)	0.1115(9)	0.115(1)	0.115(1)
*D*_JK_/kHz	−0.11	−0.147(3)	−0.143(3)	−0.138(1)	−0.140(2)	−0.141(1)
*D*_K_/kHz	0.03	0.035(2)	[0.035] ^4^	[0.035]	[0.035]	[0.035]
*d*_1_/kHz	0.04	0.0557(5)	[0.0557]	[0.0557]	[0.0557]	[0.0557]
*d*_2_/Hz	−6.6	−6.3(3)	[−6.3]	[−6.3]	[−6.3]	[−6.3]
*σ*/kHz		2.4	2.7	2.1	2.4	2.8
*N* ^5^		152	62	110	80	87
*P*_cc_/uÅ^2^	45.75	45.1961(1)	45.1821(7)	45.2522(4)	45.2435(6)	45.2020(6)

^1^ Ground-state rotational constants (*A*_0_, *B*_0_, *C*_0_) obtained by correcting the rDSDjun equilibrium values (see Table 1) with vibrational corrections at the B3 level. ^2^ Quartic centrifugal distortion constants (*D*_J_, *D*_JK_, *D*_K_, *d*_1_, *d*_2_) calculated at the B3 level. ^3^ Standard error in parenthesis in unit of the last digit. ^4^ Values in square brackets are fixed at those of the parent species. ^5^ Number of fitted transitions.

## Supplementary Materials

The following are available online, Table S1: Computed revDSD-PBEP86-D3(BJ)/jun-cc-pVTZ structures of the most stable TFAP and AP complexes with water: the first four isomers of TFAP-W and the first two isomers of AP-W in the principal inertia system. Table S2: TFAP-H_2_O measured rotational transitions of isomer *I*. Table S3: TFAP-H_2_^18^O measured rotational transitions of isomer *I*. Table S4: TFAP-D_2_O measured rotational transitions of isomer *I*. Table S5: TFAP-HOD measured rotational transitions of isomer *I*. Table S6: TFAP-DOH measured rotational transitions of isomer *I*. Table S7: NBO analysis for the TFAP-W (isomer *I* and *II*) and AP-W (isomer *I* and *II*) complexes. Table S8: SAPT analysis for the isomer *I* of TFAP-W and for the isomer *I* of AP-W. Table S9: Semi-experimental equilibrium structure (*r*^SE^) of the isolated TFAP monomer and of the isomer *I* of TFAP-W.
