# Supplementary material for: Rotational Spectroscopy Meets Quantum Chemistry for Analyzing Substituent Effects on Non-Covalent Interactions: The Case of the Trifluoroacetophenone-Water Complex
Source: Molecules. 2020 Oct 23;25(21):4899. doi: 10.3390/molecules25214899 (PMC7660205; doi:10.3390/molecules25214899)
Supplement: Supplementary file 1 [file molecules-25-04899-s001.pdf]

Supplementary Materials

Rotational Spectroscopy meets Quantum Chemistry for Analyzing Substituent Effects on Non-Covalent Interactions: the Case of the Trifluoroacetophenone-Water Complex

Juncheng Lei, Silvia Alessandrini, Junhua Chen, Yang Zheng, Lorenzo Spada, Qian Gou, Cristina Puzzarini, and Vincenzo Barone

Table of Contents

Pages S2-S7.	Table S1: Computed revDSD-PBEP86-D3(BJ)/jun-cc-pVTZ structures of the most stable TFAP and AP complexes with water: the first four isomers of TFAP-W and the first two isomers of AP-W in the principal inertia system.
Pages S8-S11.	Table S2: TFAP-H_2O measured rotational transitions of isomer *I*.
Pages S12-S13.	Table S3: TFAP-$H_2{}^{18}O$ measured rotational transitions of isomer *I*.
Pages S14-S16.	Table S4: TFAP-D_2O measured rotational transitions of isomer *I*.
Pages S17-S18.	Table S5: TFAP-HOD measured rotational transitions of isomer *I*.
Pages S19-S20.	Table S6: TFAP-DOH measured rotational transitions of isomer *I*.
Pages S21-S26.	Table S7: NBO analysis for the TFAP-W (isomers *I* and *II*) and AP-W (isomers *I* and *II*) complexes.
Page S27.	Table S8: SAPT analysis for the isomer *I* of TFAP-W and for the isomer *I* of AP-W.
Page S28.	Table S9: Semi-experimental equilibrium structure (r^{SE}) of the isolated TFAP monomer and of the isomer *I* of TFAP-W.

Table S1: Computed revDSD-PBEP86-D3(BJ)/jun-cc-pVTZ structures of the most stable TFAP and AP complexes with water: the first four isomers of TFAP-W and the first two isomers of AP-W in the principal inertia system.

Table S1.1: The isomer *I* of TFAP-W.

	X / Å	*Y* / Å	*Z* / Å
C	3.109740	-0.032045	0.000000
C	3.209316	-1.423900	0.000000
C	2.057288	-2.207153	0.000000
C	0.803685	-1.605408	0.000000
C	0.699433	-0.207074	0.000000
C	1.863060	0.577795	0.000000
H	4.004179	0.578381	0.000000
H	4.184157	-1.897026	0.000000
H	2.133388	-3.287379	0.000000
H	-0.078869	-2.228911	0.000000
H	1.786968	1.657122	0.000000
C	-0.602041	0.493570	0.000000
O	-0.736018	1.700384	0.000000
C	-1.905472	-0.354780	0.000000
F	-1.962298	-1.143409	-1.085810
F	-1.962298	-1.143409	1.085810
F	-2.978550	0.421202	0.000000
O	1.171533	3.898590	0.000000
H	0.377830	3.347326	0.000000
H	0.851197	4.803766	0.000000

Table S1.2: The isomer *II* of TFAP-W.

	X / Å	*Y* / Å	*Z* / Å
C	3.102154	1.581412	0.000000
C	3.831915	0.391161	0.000000
C	3.171626	-0.835188	0.000000
C	1.781086	-0.881292	0.000000
C	1.045887	0.310937	0.000000
C	1.715706	1.543065	0.000000
H	3.616368	2.534442	0.000000
H	4.914980	0.421070	0.000000
H	3.738597	-1.757735	0.000000
H	1.284429	-1.841295	0.000000
H	1.131414	2.454363	0.000000
C	-0.432185	0.349529	0.000000
O	-1.078739	1.376335	0.000000
C	-1.213326	-0.993481	0.000000
F	-0.911221	-1.722236	-1.085256
F	-0.911221	-1.722236	1.085256
F	-2.523288	-0.780488	0.000000
O	-3.964771	2.218790	0.000000
H	-4.491215	1.415546	0.000000
H	-3.052367	1.903521	0.000000

Table S1.3: The isomer *III* of TFAP-W.

	X / Å	*Y* / Å	*Z* / Å
C	3.346109	0.756029	-0.064504
C	3.467013	-0.631877	0.036300
C	2.328171	-1.428321	0.118989
C	1.061954	-0.849105	0.102762
C	0.938891	0.541952	-0.000247
C	2.088882	1.340976	-0.083372
H	4.231979	1.375868	-0.129164
H	4.449327	-1.089291	0.049212
H	2.417600	-2.504899	0.194476
H	0.198273	-1.495884	0.169827
H	1.972588	2.414381	-0.162754
C	-0.365805	1.242552	-0.031649
O	-0.490725	2.444671	-0.112316
C	-1.668287	0.395895	0.040860
F	-1.741291	-0.465808	-0.996142
F	-1.718643	-0.325699	1.171361
F	-2.743955	1.166612	-0.001901
O	-1.158220	-3.410906	-0.180987
H	-1.787742	-2.821245	-0.607438
H	-1.683123	-3.904893	0.455004

Table S1.4: The isomer *IV* of TFAP-W.

	X / Å	*Y* / Å	*Z* / Å
C	-2.946298	1.065071	-0.404812
C	-3.303644	0.217542	0.645265
C	-2.318239	-0.437853	1.381385
C	-0.973697	-0.258760	1.066404
C	-0.612997	0.589195	0.011348
C	-1.607395	1.254166	-0.717976
H	-3.712187	1.570996	-0.979252
H	-4.348662	0.066689	0.887749
H	-2.595026	-1.093041	2.197932
H	-0.221932	-0.777155	1.645187
H	-1.309541	1.902773	-1.531860
C	0.793620	0.822004	-0.401975
O	1.132528	1.665783	-1.199768
C	1.896640	-0.089617	0.203022
F	2.000335	0.097488	1.529341
F	1.602346	-1.388935	-0.003009
F	3.075818	0.154362	-0.342246
O	-1.357512	-2.512483	-1.542727
H	-1.696364	-1.867081	-0.914120
H	-0.422447	-2.576754	-1.327178

Table S1.5: The isomer *I* of AP-W.

	X / Å	*Y* / Å	*Z* / Å
C	-2.434055	1.343887	0.000000
C	-3.247000	0.208997	0.000000
C	-2.672400	-1.059635	0.000000
C	-1.286397	-1.196045	0.000000
C	-0.466347	-0.063007	0.000000
C	-1.051940	1.208968	0.000000
H	-2.881208	2.330632	0.000000
H	-4.325424	0.314704	0.000000
H	-3.301593	-1.941438	0.000000
H	-0.850767	-2.186985	0.000000
H	-0.404853	2.076801	0.000000
C	1.023615	-0.165588	0.000000
O	1.707100	0.850480	0.000000
C	1.659739	-1.534028	0.000000
H	1.341754	-2.096634	0.881087
H	1.341754	-2.096634	-0.881087
H	2.743012	-1.437016	0.000000
O	4.503625	0.300728	0.000000
H	3.599673	0.655581	0.000000
H	5.076750	1.070815	0.000000

Table S1.6: The isomer *II* of AP-W.

	X / Å	*Y* / Å	*Z* / Å
C	1.397528	-1.747192	0.000000
C	2.635206	-1.102963	0.000000
C	2.695347	0.288659	0.000000
C	1.519868	1.034066	0.000000
C	0.274409	0.394250	0.000000
C	0.222093	-1.006246	0.000000
H	1.348323	-2.829274	0.000000
H	3.549625	-1.684460	0.000000
H	3.654337	0.792505	0.000000
H	1.580263	2.114802	0.000000
H	-0.735373	-1.509703	0.000000
C	-0.997001	1.174972	0.000000
O	-2.087375	0.620224	0.000000
C	-0.923363	2.684385	0.000000
H	-0.387038	3.042688	0.881636
H	-0.387038	3.042688	-0.881636
H	-1.934825	3.084056	0.000000
O	-3.023632	-2.084522	0.000000
H	-2.845625	-1.131671	0.000000
H	-3.980507	-2.161080	0.000000

Table S2: TFAP-H_2O isomer *I*: measured rotational transitions (frequency values, ν, in MHz; residuals, $\Delta\nu$, in kHz).

$N°$	J	K_a	K_c	J'	K'_a	K'_c	ν	$\Delta\nu$
1	7	0	7	6	1	6	5716.0431	-3.3
2	7	1	7	6	1	6	5716.3180	-2.0
3	7	0	7	6	0	6	5717.1578	-1.8
4	7	1	7	6	0	6	5717.4334	0.1
5	5	3	3	4	2	2	6337.2107	6.7
6	7	2	6	6	2	5	6375.1933	-0.9
7	7	1	6	6	1	5	6404.6853	-1.9
8	8	0	8	7	1	7	6485.4208	-5.5
9	8	1	8	7	1	7	6485.4956	4.2
10	8	0	8	7	0	7	6485.6939	-6.0
11	8	1	8	7	0	7	6485.7716	6.6
12	4	4	1	3	3	0	6642.3112	0.0
13	4	4	0	3	3	1	6675.8801	0.5
14	6	3	4	5	2	3	6891.5027	3.1
15	7	3	5	6	3	4	6945.2917	-1.0
16	8	2	7	7	2	6	7151.6485	-1.9
17	8	1	7	7	1	6	7161.0823	-0.6
18	7	2	5	6	2	4	7201.4147	0.5
19	9	0	9	8	1	8	7254.4623	-3.1
20	9	1	9	8	1	8	7254.4776	-2.9
21	9	0	9	8	0	8	7254.5329	2.4
22	9	1	9	8	0	8	7254.5519	6.3
23	7	4	4	6	4	3	7255.7670	1.7
24	7	5	3	6	5	2	7295.5139	-0.2
25	7	5	2	6	5	1	7357.6567	1.3
26	5	4	2	4	3	1	7568.5071	2.5
27	7	4	3	6	4	2	7667.0595	-1.2
28	7	3	4	6	3	3	7769.4761	2.3
29	8	3	6	7	3	5	7781.2370	1.8
30	5	4	1	4	3	2	7789.2924	-1.9
31	8	2	6	7	2	5	7912.2994	1.7
32	9	2	8	8	2	7	7922.5279	-1.6
33	9	1	8	8	1	7	7925.2919	0.8
34	8	3	6	7	2	5	7992.3268	2.6
35	10	0	10	9	1	9	8023.4291	-5.0
36	10	1	10	9	1	9	8023.4395	1.9
37	10	0	10	9	0	9	8023.4500	0.8
38	10	1	10	9	0	9	8023.4500	-2.7
39	8	4	5	7	4	4	8226.7521	2.0
40	6	4	3	5	3	2	8328.5212	2.4
41	8	5	4	7	5	3	8371.3777	0.4
42	5	5	1	4	4	0	8411.9489	-0.6
43	5	5	0	4	4	1	8419.7549	-1.5
44	8	5	3	7	5	2	8571.0144	-0.7
45	9	3	7	8	3	6	8580.7279	-1.4

46	9	2	7	8	2	6	8633.8234	0.8
47	10	2	9	9	2	8	8691.6587	-0.1
48	10	1	9	9	1	8	8692.4209	1.0
49	8	3	5	7	3	4	8705.0114	0.9
50	11	0	11	10	1	10	8792.3962	1.7
51	11	1	11	10	0	10	8792.3962	-2.5
52	11	0	11	10	0	10	8792.3962	-1.8
53	11	1	11	10	1	10	8792.3962	0.9
54	8	4	4	7	4	3	8913.7378	1.1
55	6	4	2	5	3	3	9085.9892	4.4
56	9	4	6	8	4	5	9134.4537	0.3
57	10	3	8	9	3	7	9360.5526	1.5
58	10	2	8	9	2	7	9379.1221	-0.3
59	6	5	2	5	4	1	9403.4491	-1.5
60	9	5	5	8	5	4	9415.9050	0.7
61	11	2	10	10	2	9	9460.3743	1.1
62	11	1	10	10	1	9	9460.5760	2.2
63	6	5	1	5	4	2	9470.9103	-2.2
64	9	3	6	8	3	5	9479.4633	1.3
65	12	0	12	11	1	11	9561.3574	-0.5
66	12	1	12	11	1	11	9561.3574	-0.7
67	12	0	12	11	0	11	9561.3574	-1.3
68	12	1	12	11	0	11	9561.3574	-1.5
69	9	5	4	8	5	3	9866.1710	-0.5
70	10	4	7	9	4	6	9983.9632	0.5
71	9	4	5	8	4	4	10040.0701	-0.7
72	11	3	9	10	3	8	10131.9919	-0.8
73	11	2	9	10	2	8	10137.9070	1.0
74	6	6	1	5	5	0	10171.4433	-1.3
75	10	3	7	9	3	6	10172.1727	0.0
76	6	6	0	5	5	1	10173.0871	-3.7
77	13	0	13	12	1	12	10330.3260	1.4
78	13	1	13	12	1	12	10330.3260	1.4
79	13	0	13	12	0	12	10330.3260	1.3
80	13	1	13	12	0	12	10330.3260	1.2
81	10	5	6	9	5	5	10406.0932	1.8
82	11	4	8	10	4	7	10791.0886	-1.7
83	11	3	8	10	3	7	10872.9642	0.6
84	12	2	10	11	2	9	10902.3948	0.7
85	10	4	6	9	4	5	10992.3499	-0.5
86	13	2	12	12	2	11	10997.7838	-1.2
87	13	1	12	12	1	11	10997.7925	-5.2
88	8	5	4	7	4	3	10998.0277	3.1
89	14	0	14	13	1	13	11099.2944	1.4
90	14	1	14	13	1	13	11099.2944	1.4
91	14	0	14	13	0	13	11099.2944	1.4
92	14	1	14	13	0	13	11099.2944	1.4
93	10	5	5	9	5	4	11151.9349	-0.3
94	7	6	2	6	5	1	11187.4587	-3.0
95	7	6	1	6	5	2	11205.0064	-2.5

96	13	2	11	12	2	10	11669.1286	1.2
97	14	1	13	13	2	12	11766.5678	1.0
98	14	2	13	13	2	12	11766.5678	0.1
99	14	1	13	13	1	12	11766.5678	-3.0
100	14	2	13	13	1	12	11766.5678	-4.0
101	11	4	7	10	4	6	11767.0775	-2.1
102	15	0	15	14	0	14	11868.2633	1.5
103	15	0	15	14	1	14	11868.2633	1.6
104	15	1	15	14	0	14	11868.2633	1.5
105	15	1	15	14	1	14	11868.2633	1.6
106	8	5	3	7	4	4	11912.6156	-1.2
107	7	7	1	6	6	0	11928.3207	-0.9
108	7	7	0	6	6	1	11928.6490	1.0
109	8	6	3	7	5	2	12160.9482	-0.4
110	8	6	2	7	5	3	12259.0308	-3.9
111	14	2	11	13	2	10	12436.6452	-2.2
112	14	2	12	13	2	11	12436.7872	3.2
113	15	1	14	14	2	13	12535.3913	0.6
114	15	2	14	14	2	13	12535.3913	0.4
115	15	1	14	14	1	13	12535.3913	-0.3
116	15	2	14	14	1	13	12535.3913	-0.5
117	16	0	16	15	0	15	12637.2296	-0.1
118	16	0	16	15	1	15	12637.2296	-0.1
119	16	1	16	15	0	15	12637.2296	-0.1
120	16	1	16	15	1	15	12637.2296	-0.1
121	8	7	2	7	6	1	12951.7736	0.8
122	8	7	1	7	6	2	12955.8989	-2.3
123	16	1	15	15	2	14	13304.2411	-1.8
124	16	2	15	15	2	14	13304.2411	-1.8
125	16	1	15	15	1	14	13304.2411	-2.0
126	16	2	15	15	1	14	13304.2411	-2.0
127	17	0	17	16	0	16	13406.1970	1.1
128	17	0	17	16	1	16	13406.1970	1.1
129	17	1	17	16	0	16	13406.1970	1.1
130	17	1	17	16	1	16	13406.1970	1.1
131	8	8	1	7	7	0	13684.5605	-1.6
132	8	8	0	7	7	1	13684.6317	7.6
133	17	1	16	16	1	15	14073.1107	-4.4
134	17	1	16	16	2	15	14073.1107	-4.3
135	17	2	16	16	1	15	14073.1107	-4.4
136	17	2	16	16	2	15	14073.1107	-4.3
137	18	0	18	17	0	17	14175.1608	0.9
138	18	0	18	17	1	17	14175.1608	0.9
139	18	1	18	17	0	17	14175.1608	0.9
140	18	1	18	17	1	17	14175.1608	0.9
141	18	1	17	17	1	16	14842.0031	2.1
142	18	1	17	17	2	16	14842.0031	2.2
143	18	2	17	17	1	16	14842.0031	2.1
144	18	2	17	17	2	16	14842.0031	2.2
145	19	0	19	18	0	18	14944.1203	-0.6

146	19	0	19	18	1	18	14944.1203	-0.6
147	19	1	19	18	0	18	14944.1203	-0.6
148	19	1	19	18	1	18	14944.1203	-0.6
149	19	1	18	18	1	17	15610.8975	1.4
150	19	1	18	18	2	17	15610.8975	1.4
151	19	2	18	18	1	17	15610.8975	1.4
152	19	2	18	18	2	17	15610.8975	1.4

Table S3: TFAP-$H_2^{18}O$ isomer *I*: measured rotational transitions (frequency values, ν, in MHz; residuals, $\Delta\nu$, in kHz).

$N°$	J	K_a	K_c	J'	K'_a	K'_c	ν	$\Delta\nu$
1	9	0	9	8	1	8	7075.6131	4.1
2	9	1	9	8	1	8	7075.6131	-0.2
3	9	0	9	8	0	8	7075.6222	-7.9
4	9	1	9	8	0	8	7075.6320	-2.3
5	9	2	8	8	2	7	7730.2346	-0.2
6	9	1	8	8	1	7	7731.3366	3.1
7	10	0	10	9	1	9	7825.4569	9.0
8	10	1	10	9	1	9	7825.4569	8.1
9	10	0	10	9	0	9	7825.4569	4.7
10	10	1	10	9	0	9	7825.4569	3.9
11	5	5	1	4	4	0	8009.8718	1.0
12	5	5	0	4	4	1	8022.4528	1.1
13	10	2	9	9	2	8	8479.9177	-2.5
14	10	1	9	9	1	8	8480.1787	-3.5
15	11	0	11	10	0	10	8575.2920	2.2
16	11	1	11	10	1	10	8575.2920	2.8
17	11	0	11	10	1	10	8575.2920	3.0
18	11	1	11	10	0	10	8575.2920	2.0
19	10	3	8	9	3	7	9137.0702	1.2
20	10	2	8	9	2	7	9144.7803	-1.2
21	11	2	10	10	2	9	9229.4922	-3.1
22	11	1	10	10	1	9	9229.5555	0.4
23	12	0	12	11	1	11	9325.1358	2.0
24	12	1	12	11	1	11	9325.1358	1.9
25	12	0	12	11	0	11	9325.1358	1.8
26	12	1	12	11	0	11	9325.1358	1.8
27	6	6	1	5	5	0	9682.9094	-1.8
28	6	6	0	5	5	1	9685.9807	1.9
29	10	3	7	9	3	6	9875.7363	2.7
30	11	3	9	10	3	8	9887.1301	2.3
31	11	2	9	10	2	8	9889.2340	-2.4
32	13	0	13	12	0	12	10074.9816	0.7
33	13	0	13	12	1	12	10074.9816	0.7
34	13	1	13	12	1	12	10074.9816	0.7
35	13	1	13	12	0	12	10074.9816	0.7
36	11	3	8	10	3	7	10580.1103	1.0
37	13	1	12	12	2	11	10728.7780	-1.5
38	13	2	12	12	2	11	10728.7780	-2.3
39	13	1	12	12	1	11	10728.7780	-5.1
40	13	2	12	12	1	11	10728.7780	-5.8
41	14	0	14	13	0	13	10824.8294	0.9
42	14	0	14	13	1	13	10824.8294	0.9
43	14	1	14	13	0	13	10824.8294	0.9
44	14	1	14	13	1	13	10824.8294	0.9
45	7	7	1	6	6	0	11352.1474	-0.9

46	7	7	0	6	6	1	11352.8505	-1.1
47	15	0	15	14	0	14	11574.6773	1.8
48	15	0	15	14	1	14	11574.6773	1.8
49	15	1	15	14	0	14	11574.6773	1.8
50	15	1	15	14	1	14	11574.6773	1.8
51	15	1	14	14	2	13	12228.2340	2.1
52	15	2	14	14	2	13	12228.2340	2.0
53	15	1	14	14	1	13	12228.2340	1.9
54	15	2	14	14	1	13	12228.2340	1.9
55	16	0	16	15	0	15	12324.5201	-1.0
56	16	0	16	15	1	15	12324.5201	-1.0
57	16	1	16	15	0	15	12324.5201	-1.0
58	16	1	16	15	1	15	12324.5201	-1.0
59	17	0	17	16	0	16	13074.3630	-1.5
60	17	0	17	16	1	16	13074.3630	-1.5
61	17	1	17	16	0	16	13074.3630	-1.5
62	17	1	17	16	1	16	13074.3630	-1.5

Table S4: TFAP-D_2O isomer *I*: measured rotational transitions (frequency values, ν, in MHz; residuals, $\Delta\nu$, in kHz).

$N°$	J	K_a	K_c	J'	K'_a	K'_c	ν	$\Delta\nu$
1	8	0	8	7	1	7	6323.7153	-0.5
2	8	1	8	7	1	7	6323.7317	-1.9
3	8	0	8	7	0	7	6323.8074	3.9
4	8	1	8	7	0	7	6323.8207	-0.6
5	8	1	7	7	2	6	6976.3303	-2.1
6	8	1	7	7	1	6	6981.4478	-0.4
7	9	1	9	8	1	8	7073.3372	4.1
8	9	0	9	8	1	8	7073.3372	7.6
9	9	0	9	8	0	8	7073.3436	-3.8
10	9	1	9	8	0	8	7073.3436	-7.3
11	9	2	8	8	2	7	7727.7169	1.9
12	9	1	8	8	1	7	7728.6679	-3.9
13	10	0	10	9	1	9	7822.9289	1.0
14	10	1	10	9	1	9	7822.9289	0.3
15	10	0	10	9	0	9	7822.9289	-2.6
16	10	1	10	9	0	9	7822.9289	-3.2
17	5	5	1	4	4	0	7978.3321	-4.1
18	9	2	7	8	2	6	8403.7081	3.0
19	10	1	9	9	2	8	8477.0722	-2.4
20	10	2	9	9	2	8	8477.1406	2.5
21	10	1	9	9	1	8	8477.3597	-1.6
22	10	2	9	9	1	8	8477.4243	-0.6
23	11	0	11	10	0	10	8572.5292	-0.5
24	11	1	11	10	1	10	8572.5292	0.0
25	11	0	11	10	1	10	8572.5292	0.2
26	11	1	11	10	0	10	8572.5292	-0.7
27	10	3	8	9	3	7	9134.1414	0.0
28	10	2	8	9	2	7	9140.8948	1.3
29	11	2	10	10	2	9	9226.4818	4.5
30	11	1	10	10	1	9	9226.5293	2.1
31	12	0	12	11	1	11	9322.1327	-1.1
32	12	1	12	11	1	11	9322.1327	-1.1
33	12	0	12	11	0	11	9322.1327	-1.2
34	12	1	12	11	0	11	9322.1327	-1.2
35	6	6	1	5	5	0	9644.5221	-1.0
36	6	6	0	5	5	1	9647.8826	-0.9
37	10	3	7	9	3	6	9865.8786	-1.4
38	11	3	9	10	3	8	9883.7745	2.0
39	11	2	9	10	2	8	9885.5766	-0.5
40	12	1	11	11	2	10	9975.8655	6.9
41	12	2	11	11	2	10	9975.8655	4.0
42	12	1	11	11	1	10	9975.8655	-6.8
43	12	2	11	11	1	10	9975.8655	-9.6
44	13	0	13	12	0	12	10071.7404	0.0
45	13	0	13	12	1	12	10071.7404	0.0

46	13	1	13	12	0	12	10071.7404	0.0
47	13	1	13	12	1	12	10071.7404	0.0
48	11	3	8	10	3	7	10573.1222	-1.8
49	13	1	12	12	2	11	10725.3005	2.3
50	13	2	12	12	2	11	10725.3005	1.8
51	13	1	12	12	1	11	10725.3005	-0.5
52	13	2	12	12	1	11	10725.3005	-1.1
53	14	0	14	13	0	13	10821.3467	-0.8
54	14	0	14	13	1	13	10821.3467	-0.7
55	14	1	14	13	0	13	10821.3467	-0.8
56	14	1	14	13	1	13	10821.3467	-0.7
57	7	7	1	6	6	0	11306.6965	1.3
58	7	7	0	6	6	1	11307.4852	3.4
59	14	1	13	13	2	12	11474.7750	-1.1
60	14	2	13	13	2	12	11474.7750	-1.2
61	14	1	13	13	1	12	11474.7750	-1.6
62	14	2	13	13	1	12	11474.7750	-1.8
63	15	0	15	14	0	14	11570.9547	1.0
64	15	0	15	14	1	14	11570.9547	1.0
65	15	1	15	14	0	14	11570.9547	1.0
66	15	1	15	14	1	14	11570.9547	1.0
67	15	1	14	14	2	13	12224.2810	-0.3
68	15	2	14	14	2	13	12224.2810	-0.3
69	15	1	14	14	1	13	12224.2810	-0.4
70	15	2	14	14	1	13	12224.2810	-0.5
71	16	0	16	15	0	15	12320.5590	0.7
72	16	0	16	15	1	15	12320.5590	0.7
73	16	1	16	15	0	15	12320.5590	0.7
74	16	1	16	15	1	15	12320.5590	0.7
75	16	1	15	15	1	14	12973.8052	-0.1
76	16	1	15	15	2	14	12973.8052	0.0
77	16	2	15	15	1	14	12973.8052	-0.1
78	16	2	15	15	2	14	12973.8052	0.0
79	17	0	17	16	0	16	13070.1610	0.3
80	17	0	17	16	1	16	13070.1610	0.3
81	17	1	17	16	0	16	13070.1610	0.3
82	17	1	17	16	1	16	13070.1610	0.3
83	17	1	16	16	1	15	13723.3396	-2.0
84	17	1	16	16	2	15	13723.3396	-2.0
85	17	2	16	16	1	15	13723.3396	-2.0
86	17	2	16	16	2	15	13723.3396	-2.0
87	18	0	18	17	0	17	13819.7606	0.4
88	18	0	18	17	1	17	13819.7606	0.4
89	18	1	18	17	0	17	13819.7606	0.4
90	18	1	18	17	1	17	13819.7606	0.4
91	18	1	17	17	1	16	14472.8876	1.6
92	18	1	17	17	2	16	14472.8876	1.6
93	18	2	17	17	1	16	14472.8876	1.6
94	18	2	17	17	2	16	14472.8876	1.6
95	19	0	19	18	0	18	14569.3578	1.4

96	19	0	19	18	1	18	14569.3578	1.4
97	19	1	19	18	0	18	14569.3578	1.4
98	19	1	19	18	1	18	14569.3578	1.4
99	19	1	18	18	1	17	15222.4354	0.1
100	19	1	18	18	2	17	15222.4354	0.1
101	19	2	18	18	1	17	15222.4354	0.1
102	19	2	18	18	2	17	15222.4354	0.1
103	20	0	20	19	0	19	15318.9480	-1.0
104	20	0	20	19	1	19	15318.9480	-1.0
105	20	1	20	19	0	19	15318.9480	-1.0
106	20	1	20	19	1	19	15318.9480	-1.0
107	20	1	19	19	1	18	15971.9863	-0.9
108	20	1	19	19	2	18	15971.9863	-0.9
109	20	2	19	19	1	18	15971.9863	-0.9
110	20	2	19	19	2	18	15971.9863	-0.9

Table S5: TFAP-HOD isomer *I*: measured rotational transitions (frequency values, ν, in MHz; residuals, $\Delta\nu$, in kHz).

$N°$	J	K_a	K_c	J'	K'_a	K'_c	ν	$\Delta\nu$
1	8	1	7	7	1	6	7036.9665	1.9
2	9	0	9	8	1	8	7129.4865	1.3
3	9	1	9	8	1	8	7129.4959	4.9
4	9	1	9	8	0	8	7129.5129	-6.1
5	9	0	9	8	0	8	7129.5129	-0.2
6	9	2	8	8	2	7	7788.1644	0.9
7	9	1	8	8	1	7	7789.5497	0.2
8	10	0	10	9	1	9	7885.0910	4.7
9	10	1	10	9	1	9	7885.0910	3.5
10	10	0	10	9	0	9	7885.0910	-1.1
11	10	1	10	9	0	9	7885.0910	-2.3
12	5	5	1	4	4	0	8114.6273	-6.5
13	5	5	0	4	4	1	8125.8716	-1.8
14	10	1	9	9	2	8	8543.5569	3.0
15	10	2	9	9	2	8	8543.6564	-2.4
16	10	1	9	9	1	8	8544.0005	-0.9
17	11	0	11	10	1	10	8640.6864	-1.6
18	11	1	11	10	0	10	8640.6864	-3.1
19	11	0	11	10	0	10	8640.6864	-2.8
20	11	1	11	10	1	10	8640.6864	-1.9
21	10	3	8	9	3	7	9204.6470	2.6
22	10	2	8	9	2	7	9214.2832	0.5
23	11	2	10	10	2	9	9298.9919	1.1
24	11	1	10	10	1	9	9299.0759	4.0
25	12	0	12	11	0	11	9396.2921	-1.7
26	12	1	12	11	1	11	9396.2921	-1.5
27	12	0	12	11	1	11	9396.2921	-1.5
28	12	1	12	11	0	11	9396.2921	-1.7
29	6	6	1	5	5	0	9810.0206	0.9
30	6	6	0	5	5	1	9812.6668	0.5
31	10	3	7	9	3	6	9959.9852	-5.4
32	11	3	9	10	3	8	9960.8507	2.2
33	11	2	9	10	2	8	9963.5829	-1.4
34	13	0	13	12	0	12	10151.8995	-2.0
35	13	1	13	12	1	12	10151.8995	-2.0
36	13	0	13	12	1	12	10151.8995	-2.0
37	13	1	13	12	0	12	10151.8995	-2.0
38	11	3	8	10	3	7	10664.5119	-4.3
39	13	1	12	12	2	11	10809.7735	4.4
40	13	2	12	12	2	11	10809.7735	3.3
41	13	1	12	12	1	11	10809.7735	-0.9
42	13	2	12	12	1	11	10809.7735	-2.0
43	14	0	14	13	0	13	10907.5088	-1.3
44	14	1	14	13	1	13	10907.5088	-1.3
45	14	0	14	13	1	13	10907.5088	-1.3

46	14	1	14	13	0	13	10907.5088	-1.3
47	7	7	1	6	6	0	11501.9209	2.7
48	7	7	0	6	6	1	11502.5074	3.2
49	14	1	13	13	2	12	11565.2329	1.0
50	14	2	13	13	2	12	11565.2329	0.8
51	14	1	13	13	1	12	11565.2329	-0.1
52	14	2	13	13	1	12	11565.2329	-0.4
53	15	0	15	14	0	14	11663.1167	-1.6
54	15	0	15	14	1	14	11663.1167	-1.6
55	15	1	15	14	0	14	11663.1167	-1.6
56	15	1	15	14	1	14	11663.1167	-1.6
57	16	0	16	15	0	15	12418.7244	-0.6
58	16	0	16	15	1	15	12418.7244	-0.6
59	16	1	16	15	0	15	12418.7244	-0.6
60	16	1	16	15	1	15	12418.7244	-0.6
61	16	1	15	15	1	14	13076.2412	0.0
62	16	1	15	15	2	14	13076.2412	0.0
63	16	2	15	15	1	14	13076.2412	0.0
64	16	2	15	15	2	14	13076.2412	0.0
65	17	0	17	16	0	16	13174.3306	1.1
66	17	0	17	16	1	16	13174.3306	1.1
67	17	1	17	16	0	16	13174.3306	1.1
68	17	1	17	16	1	16	13174.3306	1.1
69	17	1	16	16	1	15	13831.7723	1.0
70	17	1	16	16	2	15	13831.7723	1.1
71	17	2	16	16	1	15	13831.7723	1.0
72	17	2	16	16	2	15	13831.7723	1.1
73	18	0	18	17	0	17	13929.9301	-1.1
74	18	0	18	17	1	17	13929.9301	-1.1
75	18	1	18	17	0	17	13929.9301	-1.1
76	18	1	18	17	1	17	13929.9301	-1.1
77	19	0	19	18	0	18	14685.5309	1.2
78	19	0	19	18	1	18	14685.5309	1.2
79	19	1	19	18	0	18	14685.5309	1.2
80	19	1	19	18	1	18	14685.5309	1.2

Table S6: TFAP-DOH isomer *I*: measured rotational transitions (frequency values, ν, in MHz; residuals, $\Delta\nu$, in kHz).

$N°$	J	K_a	K_c	J'	K'_a	K'_c	ν	$\Delta\nu$
1	8	0	8	7	1	7	6432.8034	-5.1
2	8	1	8	7	1	7	6432.8526	1.6
3	8	0	8	7	0	7	6432.9979	0.9
4	8	1	8	7	0	7	6433.0468	7.2
5	8	1	7	7	1	6	7102.3432	-0.4
6	9	2	8	8	2	7	7859.2601	-0.9
7	9	1	8	8	1	7	7861.2183	1.4
8	5	5	1	4	4	0	8263.1768	-0.2
9	5	5	0	4	4	1	8272.5968	-0.7
10	9	2	7	8	2	6	8557.4253	3.1
11	10	2	9	9	2	8	8621.9183	0.9
12	10	1	9	9	1	8	8622.4287	1.1
13	11	0	11	10	0	10	8720.8217	-2.2
14	11	1	11	10	1	10	8720.8217	-0.6
15	11	0	11	10	1	10	8720.8217	-0.1
16	11	1	11	10	0	10	8720.8217	-2.6
17	10	3	8	9	3	7	9287.4251	1.2
18	10	2	8	9	2	7	9300.8148	-0.7
19	11	2	10	10	2	9	9384.3119	6.5
20	11	1	10	10	1	9	9384.4324	-0.4
21	12	0	12	11	0	11	9483.4789	-1.4
22	12	1	12	11	1	11	9483.4789	-1.0
23	12	0	12	11	1	11	9483.4789	-0.9
24	12	1	12	11	0	11	9483.4789	-1.5
25	6	6	1	5	5	0	9990.4783	-2.0
26	6	6	0	5	5	1	9992.5818	-0.5
27	11	3	9	10	3	8	10051.4586	-0.2
28	11	2	9	10	2	8	10055.4800	-1.8
29	10	3	7	9	3	6	10069.4935	-2.7
30	12	2	11	11	2	10	10146.7021	2.5
31	12	1	11	11	1	10	10146.7243	-6.1
32	13	0	13	12	0	12	10246.1414	0.6
33	13	1	13	12	1	12	10246.1414	0.7
34	13	0	13	12	1	12	10246.1414	0.7
35	13	1	13	12	0	12	10246.1414	0.6
36	11	3	8	10	3	7	10772.4039	-2.8
37	13	1	12	12	2	11	10909.1423	-3.6
38	13	2	12	12	2	11	10909.1423	-5.8
39	13	1	12	12	1	11	10909.1638	8.5
40	13	2	12	12	1	11	10909.1638	6.4
41	14	0	14	13	0	13	11008.8012	-1.6
42	14	1	14	13	1	13	11008.8012	-1.6
43	14	0	14	13	1	13	11008.8012	-1.6
44	14	1	14	13	0	13	11008.8012	-1.6
45	14	1	13	13	2	12	11671.6449	-0.1

46	14	2	13	13	2	12	11671.6449	-0.6
47	14	1	13	13	1	12	11671.6449	-2.2
48	14	2	13	13	1	12	11671.6449	-2.7
49	7	7	1	6	6	0	11714.7476	2.4
50	7	7	0	6	6	1	11715.1873	1.0
51	15	0	15	14	0	14	11771.4638	-1.0
52	15	1	15	14	1	14	11771.4638	-1.0
53	15	0	15	14	1	14	11771.4638	-1.0
54	15	1	15	14	0	14	11771.4638	-1.0
55	15	1	14	14	1	13	12434.1787	-0.5
56	16	0	16	15	0	15	12534.1244	-1.3
57	16	1	16	15	1	15	12534.1244	-1.3
58	16	0	16	15	1	15	12534.1244	-1.3
59	16	1	16	15	0	15	12534.1244	-1.3
60	16	1	15	15	2	14	13196.7344	-3.0
61	16	2	15	15	2	14	13196.7344	-3.0
62	16	1	15	15	1	14	13196.7344	-3.1
63	16	2	15	15	1	14	13196.7344	-3.1
64	17	0	17	16	0	16	13296.7836	-1.0
65	17	1	17	16	1	16	13296.7836	-1.0
66	17	0	17	16	1	16	13296.7836	-1.0
67	17	1	17	16	0	16	13296.7836	-1.0
68	17	1	16	16	1	15	13959.3133	0.1
69	17	1	16	16	2	15	13959.3133	0.1
70	17	2	16	16	1	15	13959.3133	0.1
71	17	2	16	16	2	15	13959.3133	0.1
72	18	0	18	17	0	17	14059.4416	0.7
73	18	1	18	17	1	17	14059.4416	0.7
74	18	0	18	17	1	17	14059.4416	0.7
75	18	1	18	17	0	17	14059.4416	0.7
76	18	1	17	17	1	16	14721.9045	3.9
77	18	1	17	17	2	16	14721.9045	3.9
78	18	2	17	17	1	16	14721.9045	3.9
79	18	2	17	17	2	16	14721.9045	3.9
80	19	0	19	18	0	18	14822.0939	-0.2
81	19	1	19	18	1	18	14822.0939	-0.2
82	19	0	19	18	1	18	14822.0939	-0.2
83	19	1	19	18	0	18	14822.0939	-0.2
84	19	1	18	18	1	17	15484.4965	0.8
85	19	1	18	18	2	17	15484.4965	0.9
86	19	2	18	18	1	17	15484.4965	0.8
87	19	2	18	18	2	17	15484.4965	0.9

Table S7: NBO analysis for the TFAP-W (isomers *I* and *II*) and AP-W (isomers *I* and *II*) complexes.

Table S7.1: Stabilization energy contributions (in kJ·mol^{-1}) for the isomer *I* of TFAP-W.

From TFAP to W		
Donor NBO	Acceptor NBO	E(2)
BD (1) C6 - H11	BD*(1) O18 - H20	0.7
BD (1) C12 - C14	BD*(1) O18 - H19	0.6
LP (1) O13	BD*(1) O18 - H19	10.0
LP (2) O13	BD*(1) O18 - H19	10.8
From W to TFAP		
Donor NBO	Acceptor NBO	E(2)
BD (1) O18 - H19	BD*(1) C6 - H11	1.1
BD (1) O18 - H19	BD*(1) C12 - O13	0.6
LP (2) O18	BD*(1) C6 - H11	4.9
LP (2) O18	BD*(1) C12 - O13	0.4

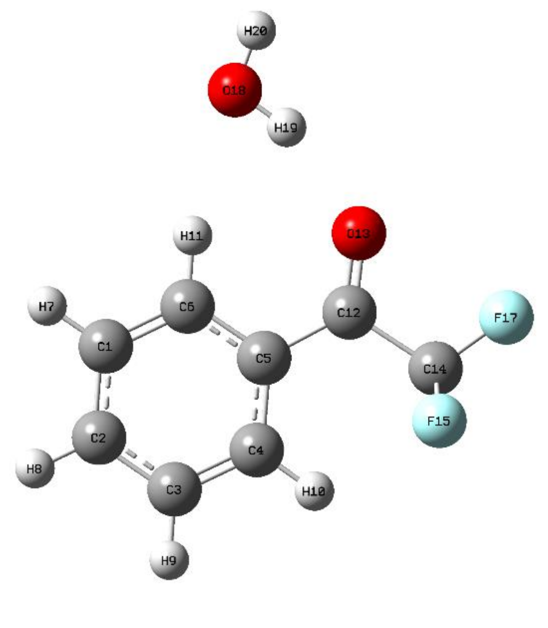

Table S7.2: NBO charges for the isomer *I* of TFAP-W together with those of the isolated TFAP and W monomers.

	TFAP-W	TFAP	W
C1	-0.200	-0.203	
C2	-0.154	-0.161	
C3	-0.208	-0.205	
C4	-0.147	-0.152	
C5	-0.180	-0.172	
C6	-0.134	-0.140	
H7	0.216	0.213	
H8	0.211	0.210	
H9	0.213	0.213	
H10	0.221	0.222	
H11	0.246	0.230	
C12	0.492	0.473	
O13	-0.535	-0.490	
C14	0.947	0.945	
F15	-0.334	-0.336	
F16	-0.334	-0.336	
F17	-0.311	-0.311	
O18	-0.956		-0.922
H19	0.486		0.461
H20	0.463		0.461

Table S7.3: Stabilization energy contributions (in kJ·mol^{-1}) for the isomer *II* of TFAP-W.

From TFAP to W		
Donor NBO	Acceptor NBO	E(2)
BD (1) C5 - C12	BD*(1) O18 - H19	0.6
LP (1) O13	BD*(1) O18 - H19	6.9
LP (2) O13	BD*(1) O18 - H19	9.0
From W to TFAP		
Donor NBO	Acceptor NBO	E(2)
BD (1) O18 - H19	BD*(1) C12 - O13	0.5
LP (1) O18	BD*(1) C12 - O13	0.3

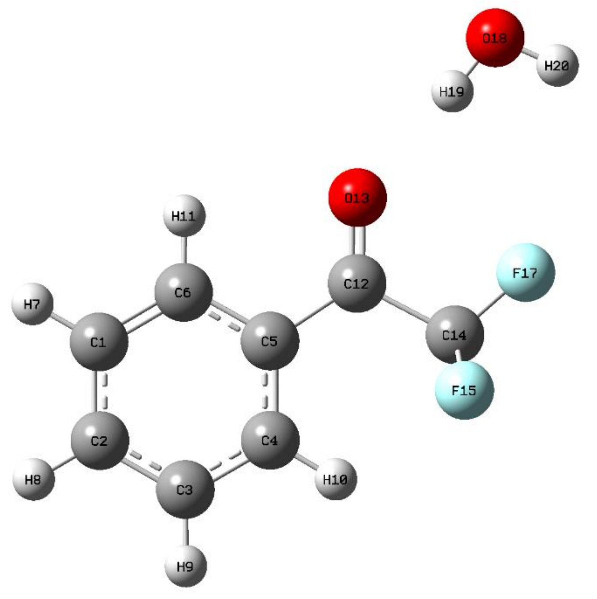

Table S7.4: NBO charges for the isomer *II* of TFAP-W together with those of the isolated TFAP and W monomers.

	TFAP-W	TFAP	W
C1	-0.202	-0.203	
C2	-0.154	-0.161	
C3	-0.205	-0.205	
C4	-0.147	-0.152	
C5	-0.177	-0.172	
C6	-0.134	-0.140	
H7	0.215	0.213	
H8	0.212	0.210	
H9	0.215	0.213	
H10	0.222	0.222	
H11	0.232	0.230	
C12	0.487	0.473	
O13	-0.523	-0.490	
C14	0.947	0.945	
F15	-0.331	-0.336	
F16	-0.331	-0.336	
F17	-0.318	-0.311	
O18	-0.943		-0.922
H19	0.481		0.461
H20	0.455		0.461

Table S7.5: Stabilization energy contributions (in $kJ \cdot mol^{-1}$) for the isomer *I* of AP-W.

From AP to W		
Donor NBO	Acceptor NBO	E(2)
BD (1) C5 - C12	BD*(1) O18 - H19	0.8
BD (1) C14 - H17	BD*(1) O18 - H20	0.5
LP (1) O13	BD*(1) O18 - H19	9.7
LP (2) O13	BD*(1) O18 - H19	28.7
**From W to AP**		
Donor NBO	Acceptor NBO	E(2)
BD (1) O18 - H19	BD*(1) C12 - O13	0.6
BD (1) O18 - H19	BD*(1) C14 - H17	0.3
LP (1) O18	BD*(1) C12 - O13	0.3
LP (1) O18	BD*(1) C14 - H17	1.8

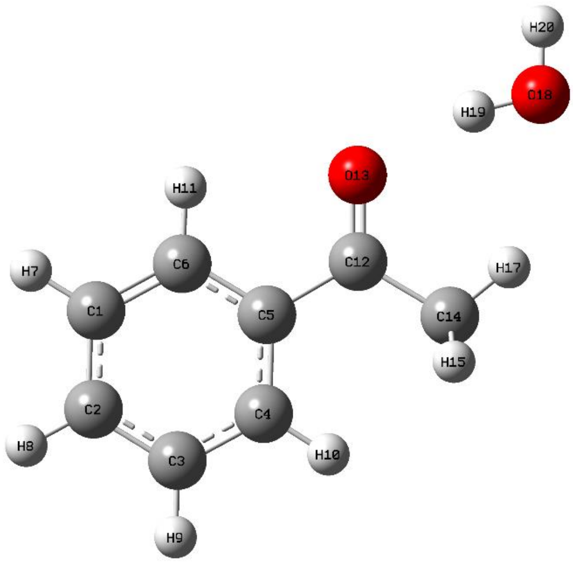

Table S7.6: NBO charges for the isomer *I* of AP-W together with those of the isolated TFAP and W monomers.

	AP-W	AP	W
C1	-0.203	-0.203	
C2	-0.172	-0.178	
C3	-0.206	-0.207	
C4	-0.167	-0.174	
C5	-0.159	-0.155	
C6	-0.146	-0.150	
H7	0.210	0.209	
H8	0.208	0.207	
H9	0.210	0.208	
H10	0.210	0.208	
H11	0.230	0.231	
C12	0.583	0.562	
O13	-0.585	-0.549	
C14	-0.692	-0.684	
H15	0.226	0.222	
H16	0.226	0.222	
H17	0.250	0.230	
O18	-0.964		-0.922
H19	0.484		0.461
H20	0.457		0.461

Table S7.7: Stabilization energy contributions (in kJ·mol^{-1}) for the isomer *II* of AP-W.

From AP to W		
Donor NBO	Acceptor NBO	E(2)
BD (1) C6 - H11	BD*(1) O18 - H20	0.6
BD (1) C12 - C14	BD*(1) O18 - H19	0.8
LP (1) O13	BD*(1) O18 - H19	14.6
LP (2) O13	BD*(1) O18 - H19	18.5
From W to AP		
Donor NBO	Acceptor NBO	E(2)
BD (1) O18 - H19	BD*(1) C6 - H11	1.2
BD (1) O18 - H19	BD*(1) C12 - O13	0.8
BD (1) O18 - H20	BD*(1) C6 - H11	0.2
LP (1) O18	BD*(2) C12 - O13	0.3
LP (2) O18	BD*(1) C6 - H11	3.6
LP (2) O18	BD*(1) C12 - O13	0.5

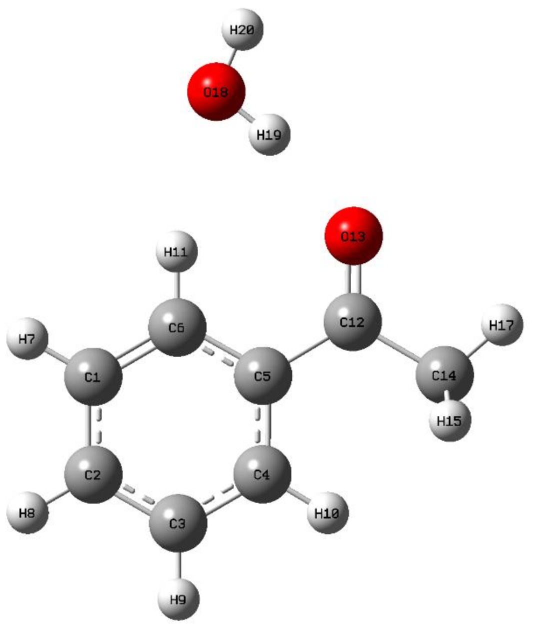

Table S7.8: NBO charges for the isomer *I* of AP-W together with those of the isolated TFAP and W monomers.

	AP-W	AP	W
C1	-0.199	-0.203	
C2	-0.170	-0.178	
C3	-0.209	-0.207	
C4	-0.168	-0.174	
C5	-0.165	-0.155	
C6	-0.143	-0.150	
H7	0.213	0.209	
H8	0.208	0.207	
H9	0.209	0.208	
H10	0.207	0.208	
H11	0.243	0.231	
C12	0.584	0.562	
O13	-0.593	-0.549	
C14	-0.683	-0.684	
H15	0.225	0.222	
H16	0.225	0.222	
H17	0.231	0.230	
O18	-0.963		-0.922
H19	0.489		0.461
H20	0.459		0.461

Table S8: SAPT analysis for the isomer *I* of TFAP-W and for the isomer *I* of AP-W.

	Electrostatics	Induction	Dispersion	Exchange	Total
TFAP-W (*I*)	-32.4	-10.7	-14.9	36.8	-21.2
AP-W (*I*)	-44.0	-15.7	-16.2	50.0	-25.9

Values in $kJ \cdot mol^{-1}$

Table S9: Semi-experimental equilibrium structure (r^{SE}) of the isolated TFAP monomer and of the isomer *I* of TFAP-W.

Table S9.1: Isolated TFAP: r^{SE} structure.

	X / Å	*Y* / Å	*Z* / Å
C	0.000000	0.000000	0.000000
C	0.000000	0.000000	1.381412
C	1.158536	0.000000	2.109474
C	1.206290	0.000000	-0.666769
H	-0.936788	0.000000	-0.543531
H	-0.938609	0.000000	1.922734
H	1.120144	0.000000	3.191763
H	1.247563	0.000000	-1.748572
C	2.407992	0.000000	0.053265
C	2.372156	0.000000	1.444822
H	3.284695	0.000000	2.024071
C	3.697277	0.000000	-0.741533
O	3.691960	0.000000	-1.945633
C	5.018610	0.000000	0.016136
F	6.048028	0.000000	-0.816225
F	5.118451	1.085570	0.802719
F	5.118451	-1.085570	0.802719

Table S9.2: TFAP-W Isomer *I*: r^{SE} structure.

	X / Å	*Y* / Å	*Z* / Å
C	0.000000	0.000000	0.000000
C	0.000000	0.000000	1.380820
C	1.158753	0.000000	2.109396
C	1.203993	0.000000	-0.671786
H	-0.935724	0.000000	-0.545042
H	-0.938606	0.000000	1.922287
H	1.121338	0.000000	3.191650
H	1.221744	0.000000	-1.753651
C	2.408198	0.000000	0.047774
C	2.370508	0.000000	1.444054
H	3.282799	0.000000	2.025836
C	3.704341	0.000000	-0.724691
O	3.732713	0.000000	-1.932028
C	5.018120	0.000000	0.048450
F	6.055154	0.000000	-0.775079
F	5.110450	1.085803	0.833731
F	5.110450	-1.085803	0.833731
O	1.781044	0.000000	-4.056453
H	2.590884	0.000000	-3.535997
H	2.066548	0.000000	-4.969339
